# Detecting Breast Cancer via Innovative Magnetic Resonance Elastography with External Vibrations to the Back

**DOI:** 10.31662/jmaj.2024-0239

**Published:** 2025-01-31

**Authors:** Emi Yamaga, Tomoyuki Fujioka, Leona Katsuta, Makiko Hayashi, Katsura Yamamuro, Yuichi Kumaki, Kumiko Hayashi, Goshi Oda, Kazunori Kubota, Ukihide Tateishi

**Affiliations:** 1 Department of Diagnostic Radiology, Institute of Science Tokyo, Tokyo, Japan; 2 Department of Radiology, Institute of Science Tokyo, Tokyo, Japan; 3 Department of Surgery, Breast Surgery, Institute of Science Tokyo, Tokyo, Japan; 4 Department of Radiology, Dokkyo Medical University Saitama Medical Center, Saitama, Japan

**Keywords:** breast cancer, magnetic resonance imaging (MRI), magnetic resonance elastography (MRE)

A woman in her 40s, with a mass detected in her right breast through screening ultrasound, underwent breast magnetic resonance elastography (MRE); the results revealed a hard, elastic mass, which was later confirmed as invasive ductal carcinoma (Figure 1, 2 and 3). The patient received hormone therapy after surgery and remains relapse-free since then. Elastography measures tissue stiffness by assessing the propagation of external vibrations and is implemented using ultrasound and magnetic resonance imaging ^(1)^. Although MRE is clinically used to assess liver cirrhosis, its application for breast imaging remains unclear ^(2)^. Previous reports required specialized devices to apply external vibrations directly to the breast for performing breast MRE ^(3), (4)^. However, we have developed a simplified approach that enables breast MRE by applying external vibrations from the back using a passive driver originally designed for liver applications (Figure 4). This method considerably contributed to a highly confident diagnosis of breast cancer.

**Figure 1.**
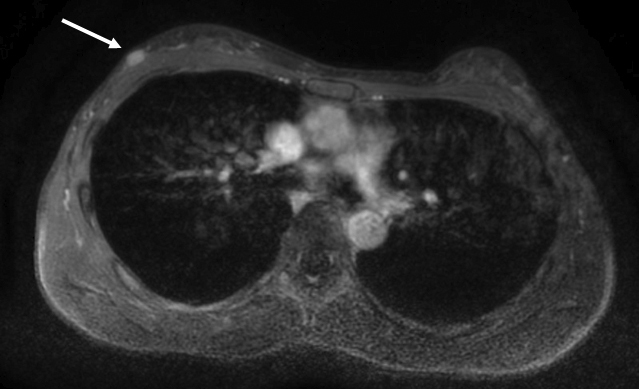
Magnetic resonance imaging (MRI) revealed an 8-mm oval mass with circumscribed margins in the upper outer quadrant of the left breast (arrow). Dynamic contrast-enhanced MRI demonstrated homogeneous enhancement with a fast washout pattern.

**Figure 2.**
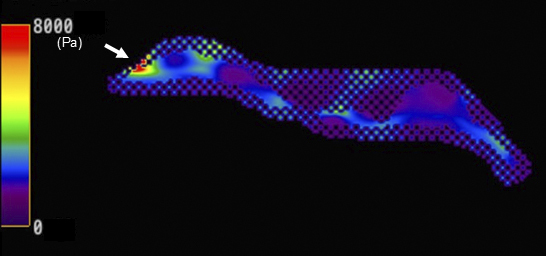
Magnetic resonance elastography (MRE) demonstrated that the mass was notably stiffer than the mammary gland (Figure 2). Areas marked with a crosshatch symbol (X) indicate regions unsuitable for stiffness measurement. Stiffness maps matching the anatomical structure of the contralateral breast were obtained, and waves propagated without interference in the MRE wave images (Figure 3). The breast magnetic resonance imaging (MRI) was obtained using a 3.0-T MRI system (Signa™ Pioneer; GE HealthCare, Milwaukee, WI, USA). Breast MRE imaging parameters are set as follows: a 16-channel breast coil, repetition time = 1000 ms, echo time = 59.2 ms, motion encoding gradient frequency = 80 Hz, vibration frequencies = 60 Hz, vibration stimulation = 70%, temporal phases = 4, number of excitations = 2, field of view = 380 mm, acquisition matrix = 64 × 64, slice thickness = 8 mm, number of slices = 13, pixel size = 5.9 × 5.9 × 8 mm^3^, and acquisition times of 69 s.

**Figure 3.**
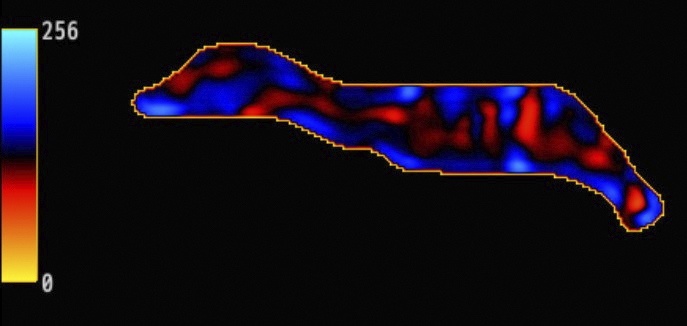
Magnetic resonance elastography (MRE) demonstrated that the mass was notably stiffer than the mammary gland (Figure 2). Areas marked with a crosshatch symbol (X) indicate regions unsuitable for stiffness measurement. Stiffness maps matching the anatomical structure of the contralateral breast were obtained, and waves propagated without interference in the MRE wave images (Figure 3). The breast magnetic resonance imaging (MRI) was obtained using a 3.0-T MRI system (Signa™ Pioneer; GE HealthCare, Milwaukee, WI, USA). Breast MRE imaging parameters are set as follows: a 16-channel breast coil, repetition time = 1000 ms, echo time = 59.2 ms, motion encoding gradient frequency = 80 Hz, vibration frequencies = 60 Hz, vibration stimulation = 70%, temporal phases = 4, number of excitations = 2, field of view = 380 mm, acquisition matrix = 64 × 64, slice thickness = 8 mm, number of slices = 13, pixel size = 5.9 × 5.9 × 8 mm^3^, and acquisition times of 69 s.

**Figure 4.**
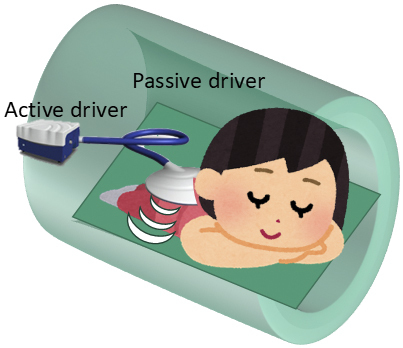
A disk-shaped passive driver is attached to the patient’s back, centered and aligned with the nipples. Air vibrations from the active driver, located in the equipment room, are transmitted to the passive driver, causing vibration. Further, this induces vibrations in the patient’s chest wall.

## Article Information

### Conflicts of Interest

None

### Sources of Funding

This work was supported by JSPS KAKENHI grant number JP21K15842.

### Acknowledgement

We used GPT-4 (https://chat.openai.com/) for Japanese to English translation and English proofreading. The generated text was reviewed, revised, and proofread by the authors. We would like to express our gratitude to “Irasutoya” for allowing us to use their illustrations in this paper.

### Author Contributions

All the authors cared for the patient, as well as wrote and approved the final manuscript.

### Approval by Institutional Review Board (IRB)

The Ethics Review Committee of the Faculty of Medicine, Institute of Science Tokyo approved this study (approval number: M2020-206). All procedures performed involving the patient were in accordance with the ethical standards of the institutional and/or National Research Committee and with the 1964 Declaration of Helsinki and its later amendments or comparable ethical standards.

### Informed Consent

Informed consent was obtained from the patient.
